# The impact of artificial intelligence on the adenoma detection rate: Comparison between experienced, intermediate and trainee endoscopists’ adenoma detection rate

**DOI:** 10.1007/s00508-025-02561-3

**Published:** 2025-06-25

**Authors:** Sebastian Bernhofer, Julian Prosenz, David Venturi, Andreas Maieron

**Affiliations:** 1 Karl Landsteiner University of Health Sciences, Dr. Karl-Dorrek-Straße 30, 3500 Krems, Austria; 2 Department of Internal Medicine 2 Gastroenterology & Hepatology, University Hospital St. Pölten, Dunant-Platz 1, 3100 St. Pölten, Austria; 3 Medical Science Research Program, Paracelsus Medical University, Strubergasse 21, 5020 Salzburg, Austria

**Keywords:** Computer-aided detection, Colonoscopy, Adenoma detection rate, Endoscopy training, Inexperienced endoscopists

## Abstract

**Background:**

Artificial intelligence (AI) is a promising tool to achieve a high adenoma detection rate (ADR). The aim of this study is to evaluate the impact of a computer-aided detection (CADe) device on the ADRs of endoscopists with different levels of expertise.

**Methods:**

Data were collected from patients who underwent colonoscopy with CADe within a 12-month period. Endoscopists were divided into three groups, a trainee group (< 500 colonoscopies), an intermediate group (500–1000 colonoscopies) and an expert group (> 2000 colonoscopies). Endoscopists with the same definition of experience without CADe support served as the control cohort. For the differences in ADR between the groups a 2-sided 95% confidence interval (CI) and odds ratios (OR) were calculated.

**Results:**

In this study 335 patients (155 females, 177 males) with a mean age 62.1 years (SD ± 16.2 years) were included in the CADe cohort. In this cohort 508 polyps were resected. The ADRs for the groups and control groups (without CADe) were as follows: 42.9% (95% CI: 28.5–57.2%) and 21.5% (95% CI: 11.3–31.8%) in the trainee group, 41.3% (95% CI: 33.5–49.0%) and 36.8% (95% CI: 27.9–45.6%) in the intermediate group and 39.8% (95% CI: 30.9–48.8%) and 33.3% (95% CI: 26.3–40.4%) in the expert group. There were no significant differences among the CADe groups when trainees were compared to experts (*p* = 0.72, OR 1.13, 95% CI: 0.58–2.16) or when intermediate endoscopists were compared to experts (*p* = 0.81, OR 1.06, 95% CI: 0.65–1.74).

**Conclusion:**

The use of AI appears to provide an opportunity to match the ADR-based quality of colonoscopy at an early stage of endoscopy training with experts.

## Introduction

Colorectal cancer (CRC) is one of the most common forms of cancer worldwide and ranks second on the list of leading causes of cancer death [1]. Prevention is key and colonoscopy has been shown to be effective through early detection and resection of neoplastic lesions [2, 3]. In order to improve the quality of colonoscopy, achieve a standardization of the procedure and make the performance of endoscopists comparable, quality parameters such as preparation quality, cecal intubation rate and adenoma detection rate (ADR) have been introduced [4]. The most important of these parameters, the ADR, which is defined by the proportion of colonoscopies in which at least one adenoma was detected compared with the total number of examinations, correlates inversely with CRC mortality [5].

The quality of colonoscopy is subject to great variability and expertise appears to be crucial for accurately identifying and removing (pre)malignant lesions [6, 7]; however, there are limited data on how the level of experience affects ADR and at what point trainees are able to perform colonoscopy on their own [8]. The use of artificial intelligence (AI) during colonoscopy has the potential to assist less experienced endoscopists and ensure a minimum level of quality at an early stage of their career. Data on the impact of computer-aided detection (CADe), a real-time AI-based polyp detection tool that assists endoscopists by highlighting suspicious mucosal areas, on the ADR of inexperienced endoscopists are promising [9–11] but the impact of AI on trainees’ ADRs has hardly been investigated.

The use of AI seems to improve colonoscopy by increasing ADR regardless of expertise. The aim of this study was to compare the ADRs of endoscopists at the very beginning of their training, intermediate endoscopists, and experts during CADe-supported colonoscopy.

## Material and methods

### AI and colonoscopy

During the relevant time period of 12 months, 1 endoscopy column (Olympus EVIS EXERA III-CV-190 (Olympus Co, Tokyo, Japan)) in the endoscopy unit was enhanced with the CADe GI-Genius™ system by Medtronic (Dublin, Ireland). All procedures were performed with a high-definition colonoscope. In order to ensure familiarization with the CADe device and exclude technical issues we defined an introduction phase lasting a few months in 2019, data from which were not included in the analysis. In general, CADe is turned on after the cecum or the terminal Ileum is reached. Location, size, and morphology according to the Paris classification [12] and histology according to the Vienna classification [13] of all removed polyps were retrieved from endoscopy and pathology reports.

### Definition of patient population

All patients aged 16 years and over having undergone colonoscopy with a CADe enhanced device were included. We categorized the patients according to indications as follows: primary screening, surveillance after polypectomy, planned polypectomy, gastrointestinal (GI) bleeding, GI disturbances, inflammatory bowel disease (IBD) surveillance and undefined reason. Exclusion criteria were poor bowel preparation (Boston bowel preparation score BBPS < 6), a history of colorectal cancer and colonoscopies with polyps that were not resected.

### Definition of CADe cohort

In this study three different groups with different levels of experience were defined. The group of endoscopists in training was defined as having performed less than 500 colonoscopies, the intermediate group as having performed more than 500 but less than 1000 colonoscopies and the expert group as having performed more than 2000 colonoscopies in their careers. All procedures were performed with a CADe GI-Genius™-enhanced colonoscope. We chose a low endoscopy volume cut-off to increase the power of the study. If more than one endoscopist was actively involved in an examination (excluding pure supervision without intervention, i.e., pointing out polyps or polypectomy), we assigned the patient to the group corresponding to the endoscopist with the higher level of training.

### Definition of control cohort

Data from patients undergoing colonoscopy before introduction of the CADe (2019) were collected to serve as a control cohort for valid interpretation of the CADe results. Colonoscopies from random months in 2017 and 2018 were included to reduce bias with partially CADe experienced staff with the aim to include a control data set of approximately equal size. Data from colonoscopies were unselectively included with the same endoscopist experience definition as in the CADe cohort.

### Outcomes and statistical analysis

The primary outcome were the ADRs of the different groups and the calculated 95% confidence intervals. The ADR was defined as the proportion of colonoscopies in which at least one adenoma was detected divided by the total number of examinations performed by the respective group. Secondary outcomes were the polyp detection rate (PDR), the advanced adenoma detection rate (AADR) and the proximal serrated polyp detection rate (PSPDR). The PDR was defined as the proportion of colonoscopies in which at least one (histologically confirmed) polyp was detected divided by the total number of examinations performed by the respective group. The AADR was defined as the proportion of colonoscopies in which at least one advanced adenoma (i.e., adenomas larger than 10 mm or with villous components or high-grade dysplasia) and the PSPDR as the proportion of colonoscopies in which at least one lesion with serrated features including hyperplastic polyps, traditional serrated adenomas and sessile serrated lesions (SSLs) proximal to the descending colon were detected, both divided by the total number of examinations. The AADR and PSPDR are reported descriptively for the entire cohort.

Results concerning analysis of colonoscopy indications, polyp characteristics including size, histological results and location are reported descriptively.

Statistical analysis was performed using IBM SPSS Statistics 23 (SPSS Inc., Armonk, NY, USA) and graphs were produced using Graphpad Prism 10 (GraphPad Software Inc., La Jolla, CA, USA).

Continuous data following a normal distribution are presented as mean and standard deviation (SD) or 95% confidence interval. Percentages are used for categorical variables. Continuous variables following a normal distribution were compared using Student’s t‑test, ordinal or nonparametric data using the Mann-Whitney test and the χ^2^- test or Fisher’s exact test (depending on the frequency) were used for categorical data.

For the differences in ADR and PDR a 2-sided 95% CI and ORs were calculated.

## Results

### Study and patient population

**Fig. 1:**
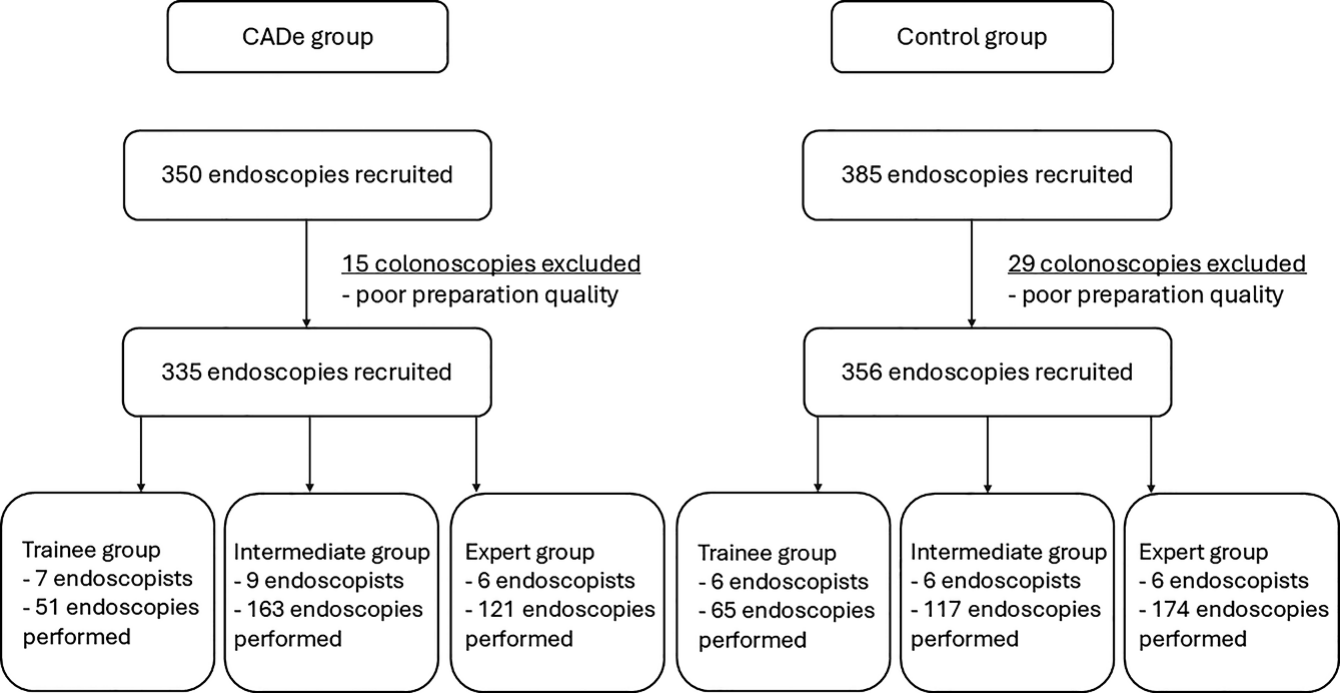
Flow chart of patient inclusion

In the CADe cohort the trainee group consisted of 7 endoscopists, the intermediate group of 9 endoscopists and the expert group of 6 endoscopists. A total number of 350 patients were included who underwent colonoscopy between January and December 2020 in the CADe-enhanced endoscopy suite. The final CADe cohort consisted of 335 patients as 15 patients were excluded because of poor preparation quality (Fig. 1). An approximately equal number of women (*n* = 155, 46.3%) and men (*n* = 177, 52.8%) were included. The mean age was 62.1 years (SD ± 16.2 years). Indications for colonoscopy were screening (*n* = 74, 22.1%), bleeding/anemia (*n* = 56, 16.7%), elective polypectomy (*n* = 51, 15.2%), surveillance (*n* = 49, 14.6%), GI disturbances (*n* = 37, 11.0%), unclassified (*n* = 37, 11.0%) and IBD (*n* = 31, 9.3%). Of note, there was a difference between the different endoscopist groups in terms of the indications. Screening and surveillance colonoscopies were more likely done by the trainee and intermediate group (*n* = 50, 67.6% and *n* = 31, 63.3% of colonoscopies in each group respectively), whereas planned polypectomies were more likely performed by the expert group (*n* = 27, 52.9% of colonoscopies in this group). Apart from the 15 excluded patients, the preparation quality was excellent (BBPS median = 9, interquartile range IQR 7–9). The cecal intubation rate (CIR) was 95.5% (*n* = 316) with no differences between the groups (trainee group 96.1%, intermediate group 96.3%, expert group 94.1%) (Table 1 and 2).

**Table 1:** CADe cohort patients’ characteristics

Colonoscopies	335
Mean age (SD), years	62.0 (16.2)
*Sex, n (%)*	
Female	155 (46.3%)
Male	177 (52.8%)
*Indications for colonoscopy, n (%)*	
Screening	74 (22.1%)
Surveillance	49 (14.6%)
Bleeding/anemia	56 (16.7%)
Elective polypectomy	51 (15.2%)
Unclassified	37 (11.0%)
IBD	31 (9.3%)
GI disturbances	37 (11.0%)
BBPS, median (IQR)	9 (7;9)

*IBD* inflammatory bowel disease, *GI* gastrointestinal, *BBPS* Boston bowel preparation scale, *IQR* interquartile range

**Table 2:** CADe cohort indications for colonoscopies

Level of training	Inexperienced endoscopists, *n* (%)	Experts, *n* (%)	Trainees, *n* (%)	Intermediate group, *n* (%)
Screening	50 (67.6)	24 (32.4)	7 (9.5)	43 (58.1)
Bleeding/anemia	40 (71.4)	16 (28.6)	13 (23.2)	27 (48.2)
Elective polypectomy	24 (47.1)	27 (52.9)	6 (11.8)	18 (35.3)
Surveillance	31 (63.3)	18 (36.7)	7 (14.3)	24 (49.0)
GI disturbances	27 (73.0)	10 (27.0)	11 (29.7)	16 (43.3)
Unclassified	24 (64.9)	13 (35.1)	3 (8.1)	21 (56.8)
IBD	18 (58.1)	13 (41.9)	4 (12.9)	14 (45.2)

*IBD* inflammatory bowel disease, *GI* gastrointestinal

Data of 385 patients were collected for the control cohort. After excluding 29 patients due to poor bowel preparation, the final control cohort consisted of 356 patients (Fig. 1). Of note, likely due to differing endoscopy reports (or policies), especially reporting of indications and bowel preparation differed in the historical control-cohort. First, mainly special indications (bleeding, IBD surveillance, polypectomy, etc.) were reported, i.e., most of the unclassified group are likely performed for screening and where thus included in screening indication subgroup analyses; for consistency this was done in both cohorts. Second, BBPS reporting was not mandatory, hence many reports only included information such as “adequate” or “inadequate”. Reported indications for colonoscopy were unclassified (*n* = 131, 36.8%), bleeding/anemia (*n* = 39, 11%), surveillance (*n* = 29, 8.1%), IBD (*n* = 29, 8.1%), GI disturbances (*n* = 26, 7.3%), elective polypectomy (*n* = 23, 6.5%) and screening (*n* = 22, 6.2%).

### Outcomes

**Fig. 2:**
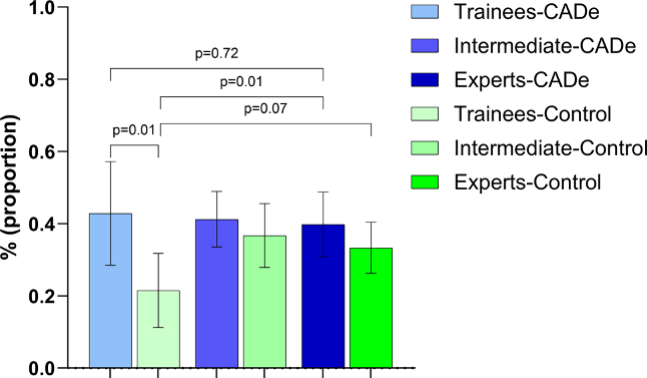
Adenoma detection rates (*ADR*) and 95% CIs of the control and CADe cohorts as well as subgroups by experience (trainee, intermediate, expert); *p*-values for comparison of trainee-control vs. experts-control, trainee-control vs. experts CADe, trainee-control vs. trainee CADe, and trainee CADe vs. expert CADe

Across the entire CADe cohort, at least 1 adenoma was detected in 134 of 327 colonoscopies (ADR 41.0%, 95% CI: 34.7–45.3%). The ADRs for the CADe groups were: 42.9% (95% CI: 28.5–57.2%) in the trainee group, 41.3% (95% CI: 33.5–49.0%) in the intermediate group and 39.8% (95% CI: 30.9–48.8%) in the expert group. No significant differences in ADR were found between the trainee and expert groups (*p* = 0.72, OR 1.13, 95% CI: 0.58–2.16), the intermediate and expert groups (*p* = 0.81, OR 1.06, 95% CI: 0.65–1.74) as well as the inexperienced group (combination of trainees and intermediate endoscopists) compared to the expert group (*p* = 0.75, OR 1.08, 95% CI: 0.68–1.69) (Fig. 2). In the CADe subgroup of patients with the indication screening, the mean ADR was 45.9% (95% CI: 36.4–55.4); in the inexperienced group the ADR was higher than in the expert group, 51.4% (95% CI: 39.6–63.2) and 35.1% (95% CI: 19.0–51.3), respectively.

A total of 508 histologically confirmed polyps (adenomas, SSLs, hyperplastic polyps) were resected in the CADe cohort. The overall PDR was 65.1% (95% CI: 59.9–70.2%). The PDRs between the inexperienced group (PDR 65.4%, 95% CI: 59.0–71.8) and the expert group (PDR 64.5%, 95% CI: 55.8–73.1) were comparable.

**Fig. 3:**
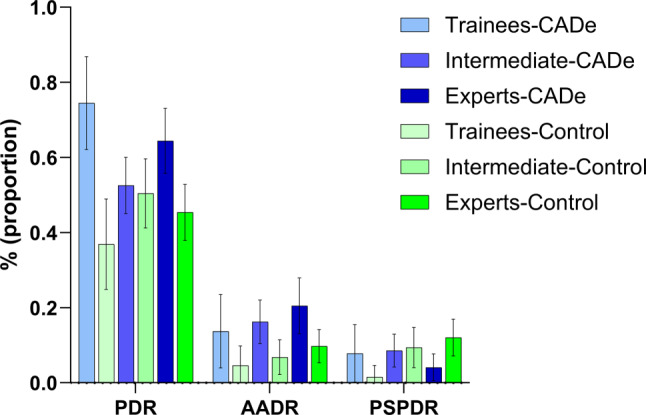
Quality indicators of different groups (trainees, intermediate endoscopists, experts), percentage with 95% CI. *PDR* polyp detection rate, *AADR* advanced adenoma detection rate, *PSPDR* proximal serrated polyp detection rate

The overall AADR was 16.8% (95% CI: 12.7–20.9%) and PSPDR 6.8% (95% CI: 4.1–9.6%) in the CADe cohort. As expected, the AADR was higher in the expert group (AADR 20.5%, 95% CI: 13.1–27.9) than in the other two groups combined (AADR 14.8%, 95% CI: 9.9–19.6). Interestingly, the opposite was true for PSPDR, expert group (PSPDR 4.1%, 95% CI: 0.5–7.7) compared to the trainee and intermediate group (PSPDR 8.4%, 95% CI: 4.7–12.2) (Table 3 and Fig. 3).

**Table 3:** Baseline characteristics control and CADe

	Control cohort			CADe cohort			
	Inexperienced	Experts	*p*-value	Inexperienced	Experts	*p*-value	*p*-value*
Sex (% male)	53.3%	45.4%	0.14	49.5%	60%	0.07	0.31
Age, years (mean)	63.9	63.0	0.55	60.9	62.2	0.53	0.16
PDR	45.6%	45.4%	0.97	65.4%	64.5%	0.86	< 0.01
ADR	31.3%	33.3%	0.69	41.6%	39.8%	0.75	0.02
AADR	6.0%	9.8%	0.19	14.8%	20.5%	0.18	< 0.01
PSPDR	6.6%	12.1%	0.08	8.4%	4.1%	0.14	0.25
BBPS median	n/a⁺	n/a⁺	n/a⁺	9	9	0.37	n/a⁺
BBPS > 5	92.5%		–	95.7%		–	0.06
Indication screening and unclassified	43.0%			33.1%			< 0.01^#^

Inexperienced = trainees + intermediate endoscopists

*p-value of comparison overall control cohort vs. CADe cohort

*PDR* polyp detection rate, *ADR* adenoma detection rate, *AADR* advanced adenoma detection rate, *PSPDR* proximal serrated polyp detection rate, *BBPS* Boston bowel preparation scale, *n/a* not available

⁺BBPS reporting incomplete as not mandatory in 2017–2018

#Screening and unclassified vs. other

In the control groups the ADRs were: 21.5% (95% CI: 11.3–31.8%) in the trainee group, 36.8% (95% CI: 27.9–45.6%) in the intermediate group and 33.3% (95% CI: 26.3–40.4%) in the expert group. The ADR was statistically significantly better in the CADe cohort than the control cohort (41.0% vs. 32.3%, *p* = 0.02). In the subgroup of trainees, the difference in ADR was even greater (CADe trainees 42.9% vs. control trainees 21.5%, *p* = 0.01) (Fig. 2). Statistically significant differences were also observed in the parameters PDR (CADe cohort 65.1% vs. control cohort 45.5%, *p* < 0.01) and AADR (CADe cohort 16.8% vs. 7.9%, *p* < 0.01) when comparing the overall cohorts (Table 3 and Fig. 3).

### Polyp characteristics in the CADe-cohort

A total of 632 polypoid lesions were removed, of which 592 were examined histologically. Of these lesions, 13 were invasive colorectal carcinomas, 71 did not correspond to polyps (normal mucosa, regenerative mucosa or inflammatory polyps) and 508 were polyps (adenomas, SSLs, hyperplastic polyps). Most polyps were adenomas (*n* = 304, 48.1%), most of which had low-grade dysplasia (*n* = 279) as opposed to high-grade dysplasia (*n* = 24). Most of the adenomas showed tubular histology (*n* = 259), villous features were found in 44 polyps. Hyperplastic polyps and SSLs accounted for 167 and 37 polyps, respectively. A total of 77 advanced adenomas were resected.

Most polyps were smaller than 5 mm (*n* = 308, 48.7%), followed by polyps between 5 and 10 mm (*n* = 164, 25.9%) and polyps greater than 10 mm (*n* = 44, 7.0%). The size of 116 polyps was not reported by the endoscopists.

In the rectosigmoid 186 polyps were detected, corresponding to 29.4% of all resected polyps, most of which were hyperplastic polyps (*n* = 103, 55.4%). No SSLs were found in the rectosigmoid. In the rest of the colon serrated lesions accounted for 31.4%, in more detail 64 hyperplastic polyps and 37 SSLs were found.

## Discussion

In this retrospective single center study exploring the effects of CADe on colonoscopy quality of inexperienced endoscopists (trainees and intermediate endoscopists) compared to experts, the ADRs between these groups were comparable (41.6% vs. 39.8%) and statistically significantly better than a corresponding control group without CADe support (CADe trainees 42.9% vs. control trainees 21.5%, *p* = 0.01).

There are a number of studies that have already shown an increase in ADR and PDR to varying degrees with AI [9, 10, 14–19]. In a systematic review and meta-analysis by Barua et al. [15] the ADR increased by 10.3%, from 19.3% without CADe to 29.6% with AI. Another systematic review and meta-analysis by Hassan et al. [19] found an even greater increase of 44%; however, few studies have focused on investigating the effects of CADe on inexperienced endoscopists or even trainees. A large study that looked at the specific impact of CADe on less experienced endoscopists was the AID-2 trial [9]. In this study, a 22% increase in ADR was observed when CADe was used. In a post hoc analysis comparing these results with the results of AID‑1, which investigated the effect of CADe on experienced endoscopists, the endoscopist’s level of experience had no effect on the improvement of ADR and ADR. In this study, the definition of the inexperienced group was relatively broad, with a threshold of less than 2000 colonoscopies. As we expect the greatest effect of AI early in the training phase, we chose a low cut-off for the less experienced group (trainees and intermediate endoscopists). Despite choosing a threshold of less than 1000 colonoscopies, there were comparable ADRs between the inexperienced and expert groups. In all groups, CADe-supported endoscopists performed better than the corresponding endoscopists without AI support; however, this was most pronounced in the trainee group. In accordance with Repici et al. [9] a significant increase in ADR of 21.4% (95% CI 4.3–38.3) was observed in the trainee group. Our findings underline the potential of AI to improve the quality of colonoscopy in the training phase and thereby possibly enable training without the need of constant direct supervision at an early stage.

In previous studies, the increase in ADR due to CADe was largely driven by a higher detection rate of small and distal lesions [14, 20]. This seems somewhat plausible as larger polyps are more likely to be detected without AI help than smaller lesions. As smaller adenomas harbor a lower malignant potential and more hyperplastic polyps are also detected by AI, concerns were raised that the use of CADe may lead to overdiagnosis of harmless polyps and therefore the benefit of AI could be overestimated [21]. In line with this, a recent meta-analysis showed that the use of CADe does not affect AADR and leads to an increase in unnecessary removal of non-neoplastic polyps [22]. In our study, 49.2% (250/508) of all detected polyps were smaller than 5 mm and 37.8% (192/508) of all polyps were found in the rectosigmoid; however, another meta-analysis [14] found that the increase in detection of rectosigmoid polyps was also associated with a higher number of detected polyps in the proximal colon when AI was in use. In our study 48.8% (248/508) of all polyps, 58.9% (179/304) of all adenomas and 48.1% (37/77) of the advanced adenomas were found proximal to the left flexure. There was no relevant difference between the different endoscopist groups regarding the location of adenoma resection. Our data are in line with the findings of Ashat et al. [14], suggesting a relevant increase in detected adenomas throughout all colon segments.

The detection rate of advanced adenomas was relatively high at 16.8% (95% CI: 12.7–20.9%) in all CADe groups combined. In comparison, the AADR in a retrospective study including more than 200,000 screening colonoscopies was 7.72% [23]. Of course, it must be taken into account that 15.2% of the colonoscopies in our study were elective polypectomies and therefore a higher AADR is to be expected. The difference in AADR between the groups (trainee and intermediate group: AADR 14.8% vs. expert group: AADR 20.5%) could be explained not only by the different skill levels but also by the different indications as more planned polypectomies were performed by the expert group; however, our study was not adequately powered to explore granular subgroup analyses.

Recently, the proximal serrated polyp detection rate (PSPDR) has gained awareness as a quality measure for colonoscopy. It is estimated that 10–33% of all new CRC evolve via the serrated pathway, which is thought to have a faster growth rate than the classical adenoma-carcinoma sequence [24, 25]. Data on the effects of AI on PSPDR are scarce but some studies suggest an increase in PSPDR when CADe was used [19, 26, 27]. In our study, the PSPDR in the CADe cohort was 6.9%, between the minimum PSPDR of 5.0% required by the British Society of Gastroenterology and the PSPDR of 11.1%, which is assumed to be associated with a significant reduction in colorectal cancer risk after screening colonoscopy [28, 29]. Interestingly the inexperienced group performed better than the expert group (trainee and intermediate group: PSPDR 8.4% vs. expert group: PSPDR 4.1%). A possible explanation for this finding was the different indications for colonoscopy between the groups.

Limitations of this study were the unbalanced distribution of indications for colonoscopy across the groups, the small number of patients for each indication, the large number of participating endoscopists and the retrospective study design.

In summary, AI appears to offer an opportunity to improve the quality of colonoscopy at an early stage of endoscopy training by reducing the interoperator skill variability. Using AI may therefore improve training opportunities for young endoscopists while ensuring a high-quality endoscopy for patients.
